# Repeated Restraint Stress and Binge Alcohol during Adolescence Induce Long-Term Effects on Anxiety-like Behavior and the Expression of the Endocannabinoid System in Male Rats

**DOI:** 10.3390/biomedicines10030593

**Published:** 2022-03-03

**Authors:** Laura Sánchez-Marín, María Flores-López, Ana L. Gavito, Juan Suárez, Francisco Javier Pavón-Morón, Fernando Rodríguez de Fonseca, Antonia Serrano

**Affiliations:** 1 Instituto de Investigación Biomédica de Málaga (IBIMA)-Plataforma Bionand, 29590 Málaga, Spain; laura.sanchez@ibima.eu (L.S.-M.); maria.flores@ibima.eu (M.F.-L.); ana.gavito@ibima.eu (A.L.G.); juan.suarez@ibima.eu (J.S.); 2 Unidad de Gestión Clínica de Salud Mental, Hospital Regional Universitario de Málaga, 29010 Málaga, Spain; 3 Facultad de Psicología, Universidad de Málaga, 29010 Málaga, Spain; 4 Unidad de Gestión Clínica Área del Corazón, Hospital Universitario Virgen de la Victoria, 29010 Málaga, Spain; 5 Centro de Investigación Biomédica en Red de Enfermedades Cardiovasculares (CIBERCV), Instituto de Salud Carlos III, 28029 Madrid, Spain

**Keywords:** alcohol, stress, adolescence, endocannabinoid system, amygdala, medial prefrontal cortex

## Abstract

(1) Background: Negative experiences during adolescence increase the vulnerability to develop mental disorders later in life. A better understanding of the mechanisms underlying these long-term alterations could help to identify better therapeutic interventions. (2) Methods: Adolescent male Wistar rats were used to explore the effects of repeated stress and alcohol exposure on anxiety-like behaviors, plasma corticosterone levels and the gene expression of the endocannabinoid system (ECS) and other relevant signaling systems (glutamatergic, corticotropin-releasing hormone (CRH) and neuropeptide Y (NPY)) in the amygdala and the medial prefrontal cortex (mPFC). (3) Results: Overall, both stress and alcohol induced anxiety-like behaviors, but only the alcohol-exposed rats displayed increased plasma levels of corticosterone. In the amygdala, there was a general deficit in the gene expression of the ECS and increases in the mRNA levels of certain subunits of glutamate receptors. Interestingly, there were significant interaction effects between stress and alcohol on the expression of the NMDA receptor subunits. In addition, increased mRNA levels of the CRH receptor were observed in alcohol-exposed rats. In the mPFC, alcohol exposure was associated with an increase in the gene expression of the ECS. By contrast, the combination of stress and alcohol produced opposite effects. (4) Conclusions: In summary, early stress and alcohol exposure induced long-term anxiety-like behavior in male rats but different mechanisms are involved in these maladaptive changes in the brain.

## 1. Introduction

Adolescence is a crucial developmental period during the transition from childhood to adulthood. During this period of maturation, many morphological and functional changes occur, which make the adolescent brain highly vulnerable to the impact of stressful events in neuronal circuitry. In fact, repeated life stress is considered as one of the major environmental risk factors that increases vulnerability to later psychiatric disorders [1]. Thus, when stress is experienced during adolescence, it produces enduring effects on behavior, including an increased susceptibility to an early onset of problem drinking and the emergence of psychiatric disorders in adulthood, such as anxiety and substance use disorders [2,3]. Restraint is widely used as a stressor in rodent models, because this model of stress does not cause physical pain or harm to the animals [4]. Numerous preclinical studies have described the behavioral and hormonal effects of repeated restraint stress, including depressive-like behaviors, anxiety-like behaviors, cognition impairments and increased plasma corticosterone levels [for review see [5]]. However, these responses associated with repeated restraint stress are more evident in adolescents than in adult rodents [6,7]. While in adult male rodents there is a habituation of the hypothalamic-pituitary-adrenal (HPA) responses to repeated exposure to the same stressor (i.e., a homotypic stressor) [8,9], repeated stress leads to an increase in the stress-induced HPA responses in early adolescence [10,11]. These sensitized hormonal stress responses might contribute to the adolescent vulnerability to develop mental disorders in adulthood [7].

Another risk factor during adolescence to facilitate vulnerability to mental disorders later in life is alcohol consumption [12,13]. Similar to stress, the adolescent brain is highly sensitive to the harmful effects of alcohol consumption. Thus, adolescent alcohol exposure induces alterations in brain structure and function that could lead to behavioral changes, including increased anxiety, alcohol preference or cognitive deficits [for review see [14,15]]. The most common drinking pattern among adolescents is binge drinking, which is considered a serious public health concern. This problematic drinking pattern consists of consuming large quantities of alcohol in a period of approximately 2 h to reach blood ethanol concentrations (BECs) of at least 80 mg/dL [16]. Binge drinking is associated with a range of acute alcohol-related problems, but also with long-term consequences, which include an increased risk of alcohol use disorders (AUD) and other psychiatric disorders, such as anxiety and depression [17,18,19]. 

Because both stress and alcohol consumption during specific developmental periods can have harmful consequences on the adolescent brain and behavior, a better understanding of the underlying mechanisms involved in the increased vulnerability to develop AUD and/or other psychiatric disorders is necessary for better prevention and treatment strategies.

Substantial evidence has shown that stress and alcohol exposure can affect the endogenous cannabinoid system (ECS) [for review see [20,21,22]], which may be a potential therapeutic target for the treatment of psychiatric diseases. The ECS is a lipid signaling system expressed throughout the central nervous system (CNS) and peripheral tissues, which is involved in several physiological functions, including the modulation of the brain reward circuitry and emotional homeostasis [20,23]. This relevant modulatory system is composed of two major types of cannabinoid receptors (CB1R and CB2R), their endogenous ligands or endocannabinoids (the best-studied are two arachidonic acid derivatives, N-arachidonoylethanolamine (anandamide or AEA) and 2-arachidonoylglycerol (2-AG)), and the enzymes involved in synthesis, transportation and degradation of endocannabinoids [24]. 

There is now considerable evidence demonstrating that the ECS can modulate alcohol-related behaviors, as well as the stress response [for review see [20,25,26]]. Anatomically, the ECS is prominently expressed within the mesocorticolimbic areas that are important for reward and motivation, including the hippocampus, amygdala, striatum or prefrontal cortex (PFC) [27,28,29,30]. Given the importance of these circuits in the regulation of emotion and reward, and the role of the ECS in the modulation of both stress responses and alcohol-related behaviors, it is possible that negative experiences during adolescence may lead to a dysregulation of the endocannabinoid function. Thus, the combination of stressful experience with alcohol consumption during this developmental period may be associated with a dysregulated ECS that influences vulnerability to mental illness in adulthood.

Recently, we have reported that both acute restraint stress and alcohol exposure during adolescence result in an increased anxiety-like behavior and high plasma levels of corticosterone in young adult male rats, but this anxious phenotype is associated with different mechanisms in the amygdala [31]. Thus, while acute restraint stress mainly alters the expression of the corticotropin releasing hormone (CRH) receptors and some glutamate receptor subunits, intermittent alcohol exposure during adolescence results in a dysregulation of 2-AG signaling and an increase of the expression of some glutamate receptor subunits. Moreover, we do not find a synergism effect on anxiety-like behaviors following the combination of acute restraint stress and alcohol consumption [31]. For a better understanding of the isolated and interactive effects of adolescent stress and alcohol consumption, the current study examined the long-term effects of repeated restraint stress and/or alcohol exposure. To this end, adolescent male Wistar rats were exposed to repeated restraint stress for five consecutive days and/or intermittent alcohol exposure using a binge-like procedure as previously described [31] to evaluate the separate and combined effects on anxiety-like behaviors and the expression of genes encoding the main components of the ECS and some subunits of receptors of the glutamatergic system in the medial PFC (mPFC) and the amygdala of young male adult rats. These brain regions play a role in the regulation of anxiety and emotion, and in the regulation of reward and addictive-related behaviors [32]. In addition, we also analyzed the effects on the mRNA expression of other signaling systems related to anxiety and stress (e.g., CRH and neuropeptide Y (NPY)) in the amygdala of young male adult rats. 

## 2. Materials and Methods

### 2.1. Animals and Ethical Statement

Forty male Wistar rats (Charles River Laboratories, Saint-Germain-Nuelles, France) weighing 75–100 g on postnatal day (PND) 21 were pair housed in a humidity and temperature-controlled vivarium on a 12 h light/dark cycle (lights off at 19:00 h). Rats were allowed to acclimatize to the new environment for several days before any experimental procedure was performed (PND32). Water and rat chow pellets were available ad libitum.

This study was designed and conducted in accordance with the European directive 2010/63/EU for the protection of animals used for scientific purposes and the Spanish regulations for the care and use of laboratory animals (Real Decreto 53/2013 and 178/2004, Ley 32/2007 and 9/2003 and Decreto 320/2010). All protocols and procedures were approved by the Ethics and Research Committee of the Universidad de Málaga. All efforts were made to minimize animal suffering, as well as to reduce the number of animals used.

### 2.2. Experimental Design

**Figure 1:**
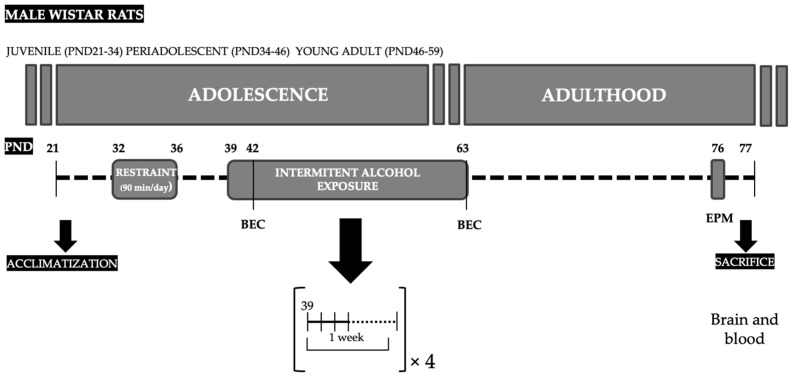
Experimental design of the study. Adolescent male rats were exposed to a repeated restraint stress (90 min/day for 5 consecutive days) from PND32 to PND36 followed by an intermittent alcohol exposure using a binge drinking procedure from PND39 to PND63. Ethanol solution was administered by intragastric gavage at doses of 3 g/kg, 4 days/week for 4 weeks. BEC values were determined 1 h after the alcohol administration at PND42 (last day of first week of alcohol exposure) and PND63 (last day of alcohol exposure). Spontaneous motor activity and anxiety-like behaviors were evaluated 2 weeks after the last administration of alcohol using the EPM. All animals were sacrificed at PND77, and brain and blood samples were collected for determinations. Abbreviations: BEC = blood ethanol concentration; EPM = elevated plus-maze; PND = postnatal day.

The experimental design is shown in Figure 1. Adolescent male rats (PND32) were randomly assigned to the experimental non-stress/stress groups (the stress (*n* = 20) and non-stress (*n* = 20) groups). Then, on PND39, rats from each group were randomly assigned to the experimental saline/alcohol subgroups (the non-stress alcohol (*n* = 10), stress alcohol (*n* = 10), non-stress saline (*n* = 10) and stress saline (*n* = 10) subgroups) following 4 weeks of intermittent exposure to alcohol or saline. 

#### 2.2.1. Repeated Restraint Stress Exposure

The animals in the stress group were subjected to 5 sessions of restraint stress. Beginning on PND32, each rat was placed individually in a flat bottom restrainer (size-adjusted) for 90 min daily for 5 consecutive days. The rats in the non-stress group were left undisturbed in their home-cages during this period. Stressed and non-stressed rats were weighed the first and the last day of restraint stress to calculate the body weight gain over the course of the stress exposure.

#### 2.2.2. Intermittent Alcohol Procedure

We used a binge drinking procedure through repeated intragastric administration of ethanol solution by gavage as previously described [31]. Rats of the stress and non-stress alcohol subgroups were administered with 3 g/kg of ethanol in a volume of 15 mL/kg (25% ethanol in saline, *v*/*v*) for 4 consecutive days and 3 days of alcohol deprivation for 4 weeks (from PND39 to PND63). Following a similar schedule and procedure to the alcohol subgroups, the rats in the stress and non-stress saline subgroups were administered with an isovolumetric equivalent of saline. The intragastric administrations by gavage were performed by a trained researcher. All the animals were weighed every week. After the adolescent alcohol/saline exposure, rats were left undisturbed in their home-cages before performing behavioral tests to evaluate anxiety-like behaviors and spontaneous motor activity two weeks later into withdrawal (i.e., a time point of withdrawal without physical symptoms).

#### 2.2.3. Determination of Blood Ethanol Concentration

All the rats in the alcohol and saline subgroups were tail bled 1 h after the last day of the first week and the last exposure to alcohol or saline. Blood samples were collected and centrifuged at 2000× *g* for 15 min to obtain plasma. Plasma was assayed for the determination of BEC using the alcohol oxidase method with an AM1 Alcohol Analyser (Analox Instruments, London, UK).

#### 2.2.4. Elevated Plus-Maze

Two weeks after the last alcohol/saline exposure (PND76), anxiety-like behaviors were evaluated using the elevated plus-maze as previously described [33]. The apparatus was made of opaque plastic and was composed of two oppositely positioned open arms (45 × 10 cm), two oppositely positioned closed arms of the same size and 50 cm high walls. The arms were connected by a central and neutral area (10 × 10 cm). The entire apparatus was elevated 75 cm above a white floor and exposed to an illumination of 70 lux. At the beginning, rats were placed in the center of the maze, facing an open arm, and were allowed to freely explore the maze for 5 min. The number of entries (an arm entry was defined as all four paws in the arm zone) and the time spent in each arm were scored using a video monitor. The number of entries into the closed arms (expressed as number of entries into the closed arms), the percent of entries into the open arms (number of entries into the open arms/total entries × 100) and the percent of time spent in the open arm (time spent in the open arms/total time × 100) were calculated.

### 2.3. Sample Collection and Brain Dissection

Twenty-four hours after performing the elevated plus-maze (PND77), rats were anesthetized with sodium pentobarbital (50 mg/kg, i.p.) and blood and brain samples were collected. The blood samples were centrifuged (2000× *g* for 15 min) and plasma aliquots were kept for further analysis. The brains were quickly removed, immediately frozen on dry ice and stored at −80 °C until mRNA analyses.

The frozen brains were placed in acrylic rat brain matrices, and 2 mm thick slices were obtained using brain matrix razor blades. The entire amygdala and the mPFC were dissected out bilaterally and samples were collected using a sample corer. Both brain regions were identified using a rat brain atlas [34]. 

### 2.4. Determination of Plasma Corticosterone Concentrations

The concentration of corticosterone in the plasma samples was determined using a commercially available enzyme-linked immunoassay kit (Abcam plc, Cambridge, UK) according to the manufacturer’s instructions.

### 2.5. RNA Isolation and RT-qPCR Analysis

Real-time PCR was used to quantify the relative mRNA levels for relevant enzymes and receptors involved in endocannabinoid signaling (receptors: CB1R (*Cnr1*), CB2R (*Cnr2*) and peroxisome proliferator-activated receptor-α (PPAR-α) (*Ppara*); synthesis enzymes: NAPE-PLD (*Napepld*), DAGL-α (*Dagla*) and DAGL-β (*Daglb*); degradation enzymes: FAAH (*Faah*) and MAGL (*Mgll*)), some receptors of glutamatergic signaling (the metabotropic receptor mGluR5 (*Grm5*); the ionotropic glutamate N-methyl D-aspartate (NMDA) receptor subunits NR1 (*Grin1*) and NR2B (*Grin2b*); and the ionotropic glutamate α-amino-3-hydroxi-5-methyl-4-isoxazolepropionic acid (AMPA) receptor subunit GluR2 (*Gria2*)) and the relative mRNA levels of neuropeptides linked to anxiety (corticotropin-releasing hormone (CRH) (*Crh*) and neuropeptide Y (NPY) (*Npy*); and receptors: CRHR1 (*Crhr1*) and NPY1r (*Npy1r*)).

Total RNA was extracted from brain samples using Trizol Reagent (Gibco BRL Life Technologies, Baltimore, MD, USA) and the concentrations were quantified using a spectrophotometer to ensure ratios of absorbance at 260 to 280 nm of 1.8–2.0. The reverse transcription was performed using the Transcriptor Reverse Transcriptase kit and random hexamer primers (Transcriptor RT; Roche Diagnostic, Mannheim, Germany). The RT-qPCR was performed using an ABI PRISMR 7300 Real-Time PCR System (Applied Biosystems, Foster City, CA, USA) and the FAM dye label format for the TaqMan Gene Expression Assays (Applied Biosystems, Foster City, CA, USA). The absolute values from each sample were normalized relative to the housekeeping gene β-actin (*Actb*), which was found to be stable among the subgroups in the amygdala and the mPFC. The relative quantification was calculated using the ΔΔCt method and normalized to the control group. Primers for the RT-qPCR were obtained based on the Applied Biosystems genome database of rat mRNA references (http://bioinfo.appliedbiosystems.com/genome-database/gene-expression.html; accessed date: 16 March 2020) (Table S1).

### 2.6. Statistical Analysis

All data for graphs and tables are expressed as the mean ± SEM. Differences in the body weight gain were statistical evaluated in rats exposed to repeated restraint stress and rats without stress using Student *t* test. The significance of differences within and between (sub)groups was evaluated using two-way analysis of variance (ANOVA) (factor 1 (f1) = “Repeated restraint stress” (levels: “non-stress” and “stress”); and factor 2 (f2) = “Adolescent alcohol exposure” (levels: “saline” and “alcohol”)). The Benjamini and Hochberg procedure to control the false discovery rate (FDR) was used as post hoc test for multiple pairwise comparisons of the subgroups when an interaction (f1 × f2) was revealed by two-way ANOVA.

The values of BEC were analyzed using two-way repeated measures ANOVA with “repeated restraint stress” (levels: “non-stress” and “stress”) as the between-subjects factor and “time of alcohol exposure” (levels: “first week of alcohol” and “last week of alcohol”) as the within-subjects factor.

Test statistic values (*t*-values and *F*-values) and degrees of freedom are indicated in the results. A *p*-value less than 0.05 was considered statistically significant for Student *t* test and ANOVA. Additionally, FDR adjusted significance levels (*q*-values) were indicated for the multiple comparisons in the interaction between factors. All statistical analyses were performed using the Graph-Pad Prism version 5.04 software (GraphPad Software, San Diego, CA, USA).

## 3. Results

In the present study, we evaluated the anxiety-like behavior, plasma corticosterone levels and gene expression of signaling systems in the amygdala and the mPFC of young male adult rats (PND77) exposed to five consecutive episodes of restraint stress and 4 weeks of intermittent alcohol during adolescence. However, other measures were collected before the sacrifice of rats.

- After the restraint stress exposure, the body weight gain in the non-stress and stress groups was as follows: 39.60 ± 0.86 g and 31.90 ± 0.98 g, respectively. Statistical analysis revealed a significant increase in body weight gain (*t*_38_ = 5.92, *p* < 0.001). However, we observed no differences in body weight gain in the rats after the intermittent alcohol exposure (data not shown).
- BEC values were determined in the plasma samples from rats exposed to alcohol at two different timepoints of the intermittent alcohol exposure. The BEC values on the last day of the first week of alcohol administration were as follows: (a) the non-stress alcohol subgroup: 132.86 ± 10.39 mg/dL; and (b) the stress alcohol subgroup: 148.99 ± 6.13 mg/dL. The BEC values on the last day of alcohol exposure were as follows: (a) the non-stress alcohol subgroup: 135.13 ± 10.94 mg/dL; and (b) the stress alcohol subgroup: 135.8 ± 11.43 mg/dL. Statistical analysis revealed no significant effects of repeated restraint stress or time of alcohol exposure on the BEC values.

### 3.1. Effects of Restraint Stress and Intermittent Alcohol on Anxiety-like Behavior

**Figure 2:**
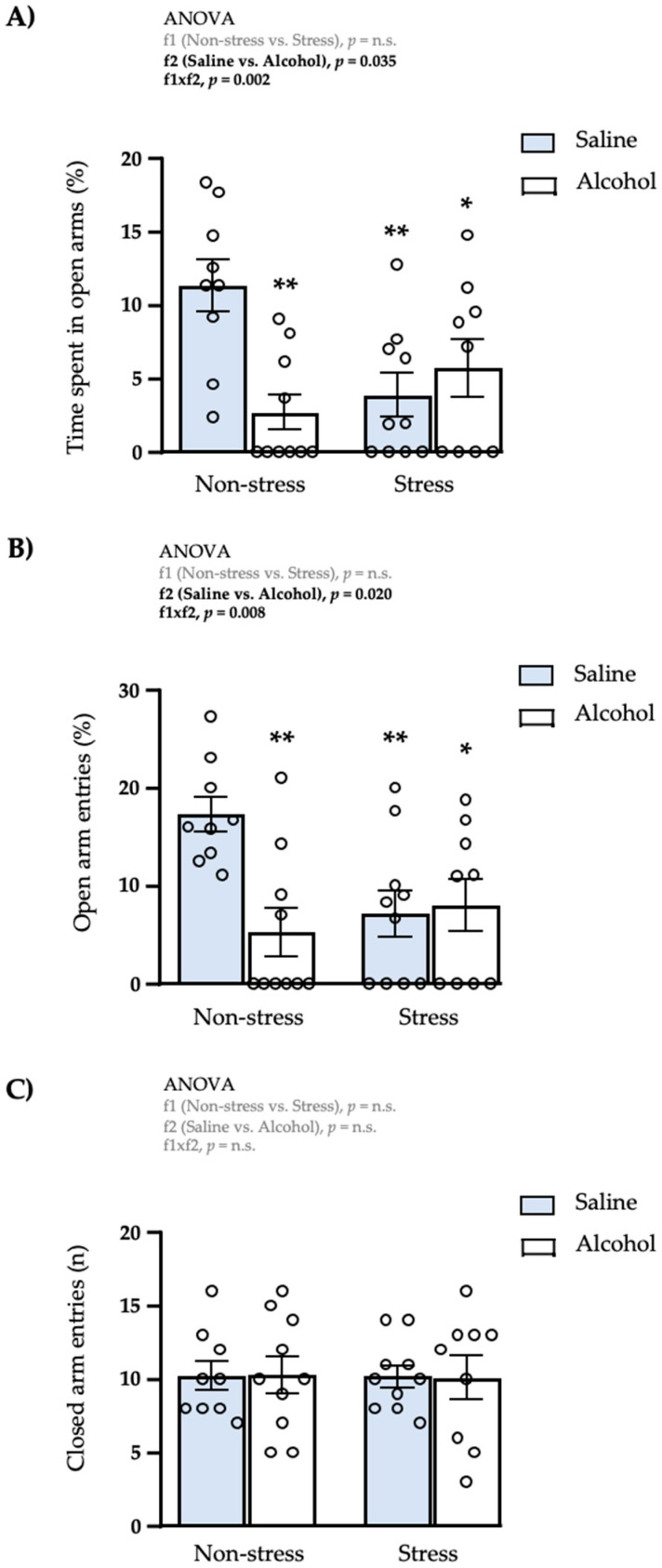
Long-term effects on anxiety-like behaviors in male rats exposed to repeated restraint stress and/or intermittent alcohol exposure during adolescence. Percentage of time spent in the open arms (**A**); percentage of open arm entries (**B**); and number of closed arm entries (**C**) in the EPM two weeks after the last alcohol administration. Male adult rats were exposed to repeated restraint stress (f1) and/or intermittent alcohol (f2) during adolescence. Bars represent the mean ± SEM (9–10 rats/subgroup). Data were analyzed by two-way ANOVA. (*) *q* < 0.05 and (**) *q* < 0.01 denote significant differences compared with the non-stress saline subgroup using the post hoc test for multiple comparisons when an interaction between factors is found (f1 × f2). *p*-values in bold denote significant main effects of factors (f1 and f2) or significant interaction (f1 × f2).

We explored the effects of repeated restraint stress and/or intermittent alcohol exposure during adolescence on anxiety-like behaviors in young adult rats. Two weeks after the last administration of alcohol (PND76), rats were tested on the elevated plus-maze to evaluate anxiety-like behaviors and spontaneous motor activity. A two-way ANOVA of the time spent in open arms revealed a significant main effect of adolescent alcohol exposure (f1: *F*_1,34_ = 4.84; *p* = 0.035), and a significant interaction between repeated restraint stress and adolescent alcohol exposure (f1 × f2: *F*_1,34_ = 11.47; *p* = 0.002) (Figure 2A). The post hoc test for multiple comparisons indicated a significant decrease in the time of open arm exploration in all subgroups compared with the non-stress saline subgroup (* *q* < 0.05 and ** *q* < 0.01).

We also evaluated anxiety-like behaviors by the number of entries in the open arms (Figure 2B). Statistical analysis revealed a main effect of adolescent alcohol exposure (f1: *F*_1,34_ = 5.98; *p* = 0.020), and a significant interaction between repeated restraint stress and adolescent alcohol exposure (f1 × f2: *F*_1,34_ = 7.85; *p* = 0.008). Similarly, the post hoc test for multiple comparisons showed a significant decrease in the number of entries into the open arms in all subgroups compared with the non-stress saline subgroup (* *q* < 0.05 and ** *q* < 0.01).

Regarding the spontaneous motor activity, we found no main effects (or interaction) of both factors in the number of entries into the closed arms (Figure 2C).

### 3.2. Effects of Restraint Stress and Intermittent Alcohol on Plasma Corticosterone Levels

**Figure 3:**
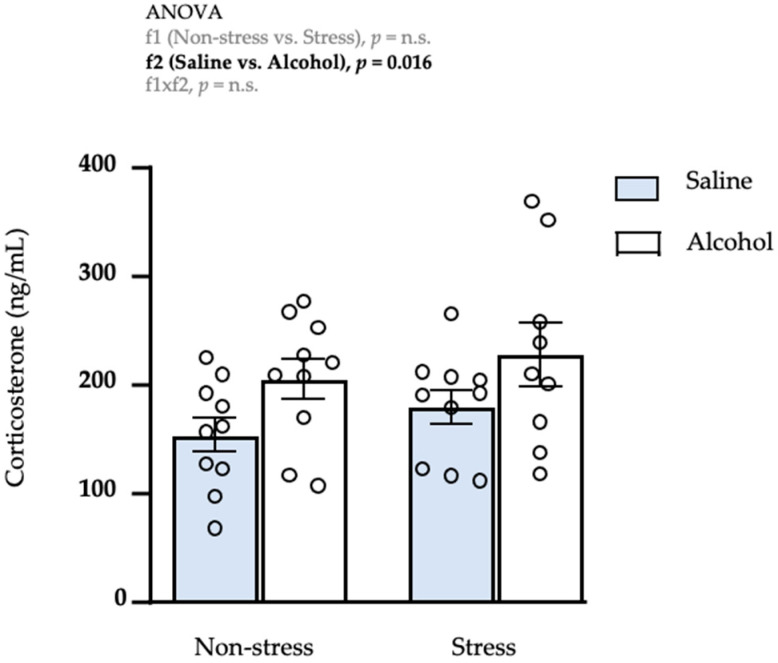
Plasma levels of corticosterone. Corticosterone was determined in male adult rats after repeated restraint stress (f1) and/or intermittent alcohol exposure (f2) during adolescence. Bars represent the mean ± SEM (9–10 rats/subgroup). Data were analyzed by two-way ANOVA. *p*-value in bold denotes significant main effect of factor 2 (f2).

We also evaluated the plasma levels of corticosterone in rats exposed to repeated restraint stress and/or intermittent alcohol during adolescence (Figure 3). A two-way ANOVA revealed a significant main effect of adolescent alcohol exposure (f2: *F*_1,35_ = 6.40; *p* = 0.016) on plasma corticosterone, but no main effect of repeated restraint stress or interaction between factors. As we can see in the figure, rats exposed to intermittent alcohol displayed higher plasma levels of corticosterone than rats exposed to saline.

### 3.3. Effects of Restraint Stress and Intermittent Alcohol on the mRNA Expression of Endocannabinoid-Signaling Genes in the Amygdala

Next, we evaluated the effects of repeated restraint stress and/or intermittent alcohol exposure during adolescence on the mRNA expression of genes related to the ECS in the amygdala.

#### 3.3.1. Receptors

**Figure 4:**
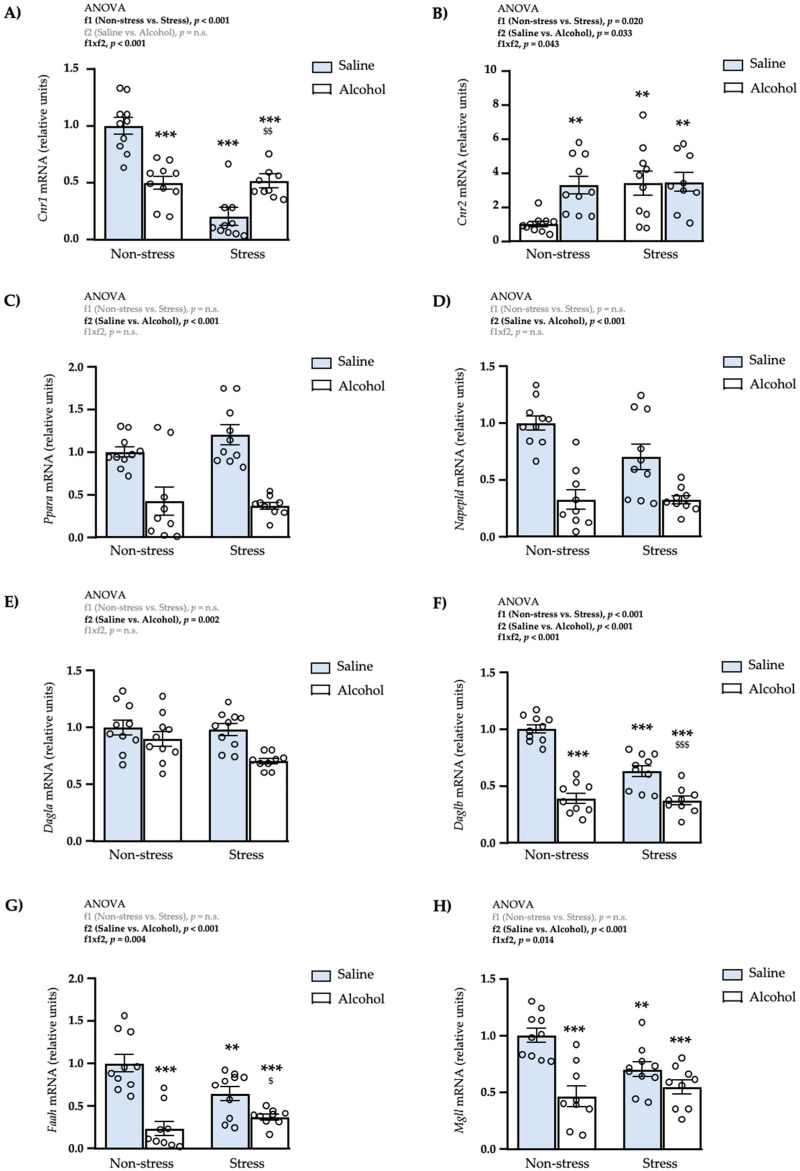
Relative mRNA expression levels of endocannabinoid-signaling genes in the amygdala of adult male rats after repeated restraint stress and/or intermittent alcohol exposure during adolescence. Relative mRNA expression of *Cnr1* (**A**); *Cnr2* (**B**); *Ppara* (**C**); *Napepld* (**D**); *Dagla* (**E**); *Daglb* (**F**); *Faah* (**G**); and *Mgll* (**H**) in male adult rats after repeated restraint stress (f1) and/or intermittent alcohol exposure (f2) during adolescence. Bars represent the mean ± SEM (9–10 rats/subgroup). Data were analyzed by two-way ANOVA. (**) *q* < 0.01 and (***) *q* < 0.001 denote significant differences compared with the non-stress saline subgroup, and (^$^) *q* < 0.05, (^$$^) *q* < 0.01 and (^$$$^) *q* < 0.001 denote significant differences compared with the stress saline subgroup using post hoc tests for multiple comparisons when an interaction between factors is found (f1 × f2). *p*-values in bold denote significant main effects of factors (f1 and f2) or significant interaction (f1 × f2).

- As shown in Figure 4A, a two-way ANOVA revealed significant main effects of repeated restraint stress (f1: *F*_1,35_ = 33.20; *p* < 0.001), and a significant interaction between repeated restraint stress and adolescent alcohol exposure (f1 × f2: *F*_1,35_ = 34.91; *p* < 0.001) on the mRNA levels of *Cnr1*. The post hoc test for multiple comparisons showed a significant decrease in the mRNA levels of this receptor in all subgroups compared with the non-stress saline subgroup (*** *q* < 0.001), and a significant increase in the stress alcohol subgroup compared with the stress saline subgroup (^$$^ *q* < 0.01).
- Regarding CB2R, the statistical analysis revealed significant main effects of repeated restraint stress (f1: *F*_1,35_ = 5.96; *p* = 0.020) and adolescent alcohol exposure (f2: *F*_1,35_ = 4.90; *p* = 0.033), and a significant interaction (f1 × f2: *F*_1,35_ = 4.41; *p* = 0.043) on the expression of *Cnr2* (Figure 4B). The post hoc test for multiple comparisons showed a significant increase in the mRNA levels of *Cnr2* in all subgroups compared with the non-stress saline subgroup (** *q* < 0.01).
- Finally, there was only one significant main effect of adolescent alcohol exposure (f2: *F*_1,34_ = 42.55; *p* < 0.001) on the mRNA levels of *Ppara* (Figure 4C). Specifically, rats exposed to intermittent alcohol displayed significantly lower levels of *Ppara* than rats exposed to saline.

#### 3.3.2. Enzymes of Synthesis

- A two-way ANOVA revealed a significant main effect of adolescent alcohol exposure on the mRNA levels of *Napepld* (f2: *F*_1,34_ = 42.85; *p* < 0.001) and the rats exposed to alcohol had significantly lower levels than rats exposed to saline (Figure 4D).
- Similar to *Napepld*, the statistical analysis showed a significant main effect of adolescent alcohol exposure on the mRNA levels of *Dagla* (f2: *F*_1,35_ = 10.82; *p* = 0.002) and the rats exposed to alcohol had significantly lower levels of *Dagla* than rats exposed to saline (Figure 4E).
- Regarding DAGL-β, there were significant main effects of repeated restraint stress (f1: *F*_1,34_ = 18.44; *p* < 0.001) and adolescent alcohol exposure (f2: *F*_1,34_ = 91.77; *p* < 0.001), and a significant interaction between both factors (f1 × f2: *F*_1,34_ = 14.85; *p* < 0.001) on the expression of *Daglb* (Figure 4F). The post hoc test for multiple comparisons showed a significant decrease in the mRNA levels of this enzyme in all subgroups compared with the non-stress saline subgroup (*** *q* < 0.001), and a significant decrease in the stress alcohol subgroup compared with the stress saline subgroup (^$$$^ *q* < 0.001).

#### 3.3.3. Enzymes of Degradation

- As shown in Figure 4G, there was a significant main effect of adolescent alcohol exposure on the mRNA levels of *Faah* (f2: *F*_1,34_ = 41.01; *p* < 0.001) and a significant interaction between both factors (f1 × f2: *F*_1,34_ = 9.48; *p* = 0.004). The post hoc test for multiple comparisons showed a significant decrease in the expression of *Faah* in all subgroups compared with the non-stress saline subgroup (*** *q* < 0.001 and ** *q* < 0.01). In addition, the stress alcohol subgroup displayed a significant decrease in *Faah* compared with the stress saline subgroup (^$^ *q* < 0.05).
- Similar to *Faah*, the statistical analysis revealed a significant main effect of adolescent alcohol exposure on the mRNA levels of *Mgll* (f2: *F*_1,34_ = 21.19; *p* < 0.001) and a significant interaction (f1 × f2: *F*_1,34_ = 6.77; *p* = 0.014) (Figure 4H). Again, the post hoc test for multiple comparisons showed a significant decrease in expression of *Mgll* in all subgroups compared with the non-stress saline subgroup (*** *q* < 0.001 and ** *q* < 0.01).

### 3.4. Effects of Restraint Stress and Intermittent Alcohol on the mRNA Expression of Glutamatergic-Signaling Genes in the Amygdala

We also evaluated the effects of repeated restraint stress and/or intermittent alcohol exposure during adolescence on the mRNA expression of some receptors involved in the glutamatergic signaling in the amygdala.

#### 3.4.1. Metabotropic Receptor

**Figure 5:**
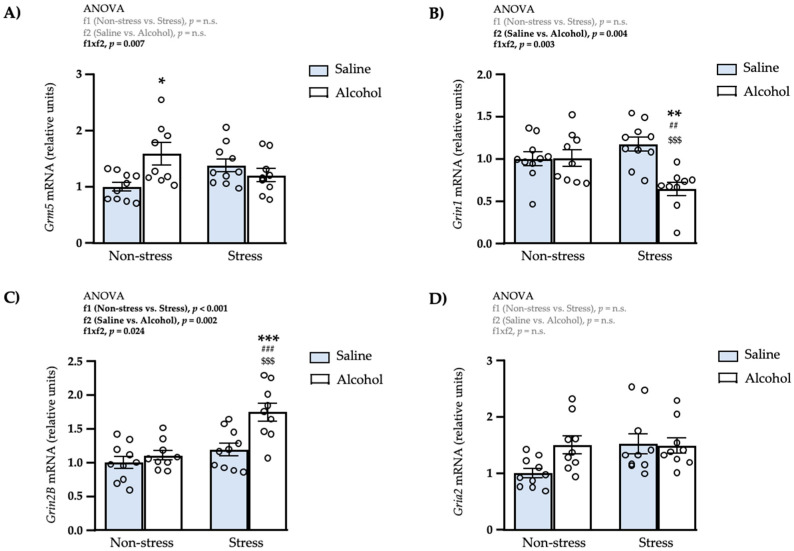
Relative mRNA expression levels of glutamatergic receptor subunits in the amygdala of adult male rats after repeated restraint stress and/or intermittent alcohol exposure during adolescence. Relative mRNA expression of *Grm5* (**A**); *Grin1* (**B**); *Grin2B* (**C**); and *Gria2* (**D**) in male adult rats after repeated restraint stress (f1) and/or intermittent alcohol exposure (f2) during adolescence. Bars represent the mean ± SEM (9–10 rats/subgroup). Data were analyzed by two-way ANOVA. (*) *q* < 0.05, (**) *q* < 0.01 and (***) *q* < 0.001 denote significant differences compared with the non-stress saline subgroup, (^$$$^) *q* < 0.001 denotes significant differences compared with the stress saline subgroup, and (^##^) *q* < 0.01 and (^###^) *q* < 0.001 denote significant differences with the non-stress alcohol subgroup using post hoc tests for multiple comparisons when an interaction between factors is found (f1 × f2). *p*-values in bold denote significant main effects of factors (f1 and f2) or significant interaction (f1 × f2).

As shown in Figure 5A, a two-way ANOVA showed a significant interaction between repeated restraint stress and adolescent alcohol exposure (f1 × f2: *F*_1,34_ = 8.33; *p* = 0.007) on the mRNA levels of *Grm5*. The post hoc test for multiple comparisons showed a significant increase in the expression of *Grm5* in the non-stress alcohol subgroup compared with the non-stress saline subgroup (* *q* < 0.05).

#### 3.4.2. NMDA Receptors

- The statistical analysis revealed one significant main effect of adolescent alcohol exposure (f2: *F*_1,34_ = 9.70; *p* = 0.004) and a significant interaction between both factors (f1 × f2: *F*_1,34_ = 10.44; *p* = 0.003) on the mRNA levels of *Grin1* (Figure 5B). The post hoc test for multiple comparisons showed that the stress alcohol group had a significant decrease in the expression of *Grin1* compared with the non-stress saline (** *q* < 0.01), non-stress alcohol (^##^ *q* < 0.01) and stress saline (^$$$^ *q* < 0.001) subgroups.
- Regarding the expression of *Grin2B* (Figure 5C), there were significant main effects of repeated restraint stress (f1: *F*_1,34_ = 18.77; *p* < 0.001) and adolescent alcohol exposure (f2: *F*_1,34_ = 11.59; *p* = 0.002), and a significant interaction between both factors (f1 × f2: *F*_1,34_ = 5.63; *p* = 0.024). In this case, the post hoc test showed that the stress alcohol subgroup had a significant increase in the expression of *Grin2B* compared with the non-stress saline (*** *q* < 0.001), non-stress alcohol (^###^ *q* < 0.001) and stress saline (^$$$^ *q* < 0.001) subgroups.

#### 3.4.3. AMPA Receptor

As shown in Figure 5D, a two-way ANOVA showed no significant main effects of repeated restraint stress and adolescent alcohol exposure or interaction between both factors.

### 3.5. Effects of Restraint Stress and Intermittent Alcohol on the mRNA Expression of Anxiety-Related Genes in the Amygdala

We explored the effects of repeated restraint stress and intermittent alcohol exposure during adolescence on the mRNA expression of many genes related to the CRH and NPY systems in the amygdala because this brain region is involved in the regulation of anxiety-like behaviors.

#### 3.5.1. CRH System

**Figure 6:**
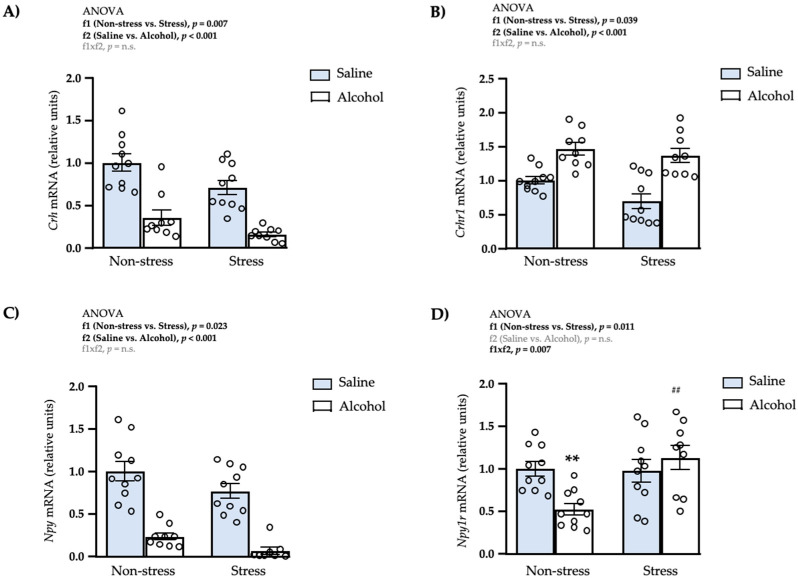
Relative mRNA expression levels of CRH and NPY systems in the amygdala of adult male rats after repeated restraint stress and/or intermittent alcohol exposure during adolescence. Relative mRNA expression of *Crh* (**A**); *Crhr1* (**B**); *Npy* (**C**); and *Npy1r* (**D**) in male adult rats after repeated restraint stress (f1) and/or intermittent alcohol exposure (f2) during adolescence. Bars represent the mean ± SEM (9–10 rats/subgroup). Data were analyzed by two-way ANOVA. (**) *q* < 0.01 denotes significant differences compared with the non-stress saline subgroup and (^##^) *q* < 0.01 denotes significant differences with the non-stress alcohol subgroup using post hoc tests for multiple comparisons when an interaction between factors is found (f1 × f2). *p*-values in bold denote significant main effects of factors (f1 and f2) or significant interaction (f1 × f2).

- There were significant main effects of repeated restraint stress (f1: *F*_1,34_ = 8.15; *p* = 0.007) and adolescent alcohol exposure (f2: *F*_1,34_ = 50.93; *p* < 0.001) on the mRNA expression of C*rh* (Figure 6A). Thus, while rats with restraint stress showed lower levels of C*rh* than non-stressed rats, rats exposed to intermittent alcohol showed lower C*rh* than rats exposed to saline.
- Regarding the mRNA expression of *Crhr1*, there were significant main effects of repeated restraint stress (f1: *F*_1,34_ = 4.89; *p* = 0.039) and adolescent alcohol exposure (f2: *F*_1,34_ = 38.47; *p* < 0.001) but no a significant interaction between factors (Figure 6B). Specifically, rats with restraint stress showed significantly lower levels of C*rhr1* than non-stressed rats, and rats exposed to alcohol showed higher levels of C*rhr1* than rats exposed to saline.

#### 3.5.2. NPY System

- A two-way ANOVA revealed significant main effects of repeated restraint stress (f1: *F*_1,33_ = 5.69; *p* = 0.023) and adolescent alcohol exposure (f2: *F*_1,33_ = 80.83; *p* < 0.001) on the mRNA levels of *Npy* (Figure 6C). Thus, rats exposed to restraint stress had significantly lower levels of *Npy* than non-stressed rats, and rats exposed to intermittent alcohol also had lower levels of *Npy* than rats with saline.
- Regarding its receptor *Npy1r*, there was a significant main effect of repeated restraint stress (f1: *F*_1,34_ = 7.26; *p* = 0.011) and a significant interaction (f1 × f2: *F*_1,34_ = 8.28; *p* = 0.007) (Figure 6D). The post hoc test for multiple comparisons showed that the non-stress alcohol subgroup had a significant decrease in the expression of *Npy1r* compared with the non-stress saline (** *q* < 0.01) and stress alcohol (^##^ *q* < 0.01) subgroups.

### 3.6. Effects of Restraint Stress and Intermittent Alcohol on the mRNA Expression of Endocannabinoid-Signaling Genes in the mPFC

Next, we evaluated the effects of repeated restraint stress and intermittent alcohol exposure on the mRNA expression of genes related to the ECS in the mPFC. 

#### 3.6.1. Receptors

**Figure 7:**
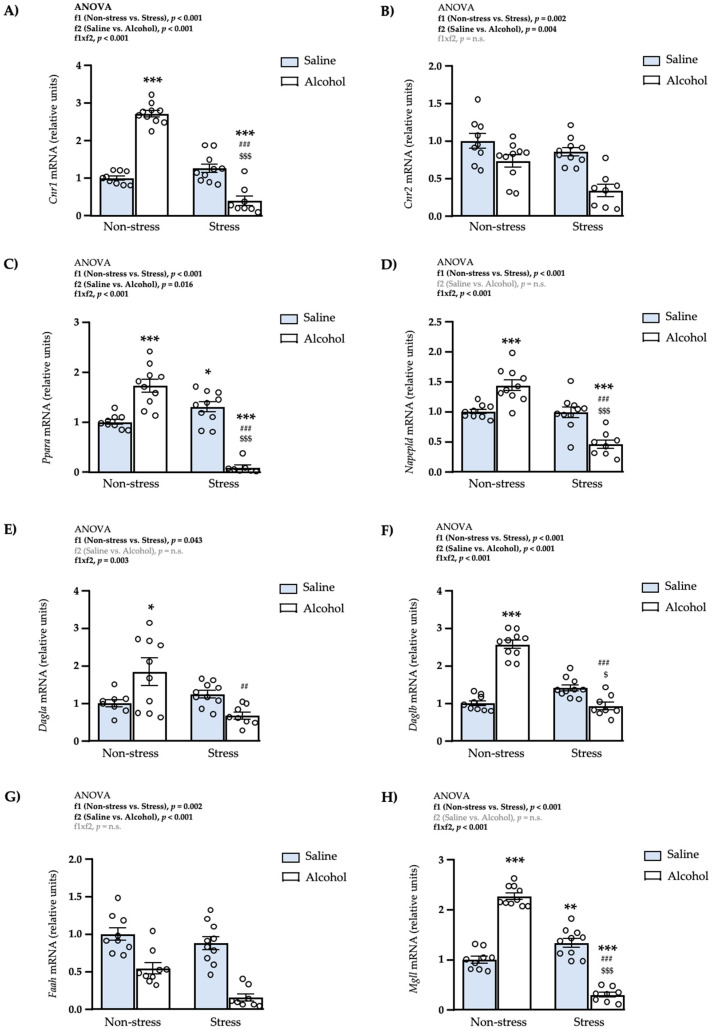
Relative mRNA expression levels of endocannabinoid-signaling genes in the mPFC of adult male rats after repeated restraint stress and/or intermittent alcohol exposure during adolescence. Relative mRNA expression of *Cnr1* (**A**); *Cnr2* (**B**); *Ppara* (**C**); *Napepld* (**D**); *DagIa* (**E**); *Daglb* (**F**); *Faah* (**G**); and *Mgll* (**H**) in male adult rats after repeated restraint stress (f1) and/or intermittent alcohol exposure (f2) during adolescence. Bars represent the mean ± SEM (8–10 rats/subgroup). Data were analyzed by two-way ANOVA. (*) *q* < 0.05, (**) *q* < 0.01 and (***) *q* < 0.001 denote significant differences compared with the non-stress saline subgroup, (^$^) *q* < 0.05 and (^$$$^) *q* < 0.001 denote significant differences compared with the stress saline subgroup, and (^##^) *q* < 0.01 and (^###^) *q* < 0.001 denotes significant differences with the non-stress alcohol subgroup using post hoc tests for multiple comparisons when an interaction between factors is found (f1 × f2). *p*-values in bold denote significant main effects of factors (f1 and f2) or significant interaction (f1 × f2).

- As shown in Figure 7A, the statistical analysis of the mRNA expression of *Cnr1* revealed significant main effects of repeated restraint stress (f1: *F*_1,33_ = 103.8; *p* < 0.001) and adolescent alcohol exposure (f2: *F*_1,33_ = 17.52; *p* < 0.001), and a significant interaction (f1 × f2: *F*_1,33_ = 161.9; *p* < 0.001). The post hoc tests showed a significant increase in the expression of *Cnr1* in the non-stress alcohol subgroup compared with the non-stress saline subgroup (*** *q* < 0.001), and a significant decrease in the stress alcohol subgroup compared with the non-stress saline (*** *q* < 0.001), non-stress alcohol (^###^ *q* < 0.001) and stress saline (^$$$^ *q* < 0.001) subgroups.
- Regarding the gene expression of CB2R, there were significant main effects of repeated restraint stress (f1: *F*_1,33_ = 11.24; *p* = 0.002) and adolescent alcohol exposure (f2: *F*_1,33_ = 24.21; *p* < 0.001) but no significant interaction between both factors (Figure 7B). Therefore, rats exposed to restraint stress had significantly lower levels of *Cnr2* than non-stressed rats, and rats exposed to intermittent alcohol had lower levels of *Cnr2* than rats exposed to saline.
- For the expression of *Ppara*, there were significant main effects of repeated restraint stress (f1: *F*_1,32_ = 47.51; *p* < 0.001) and adolescent alcohol exposure (f2: *F*_1,32_ = 6.43; *p* = 0.016) but a significant interaction was also revealed by the two-way ANOVA (f1 × f2: *F*_1,32_ = 100.2; *p* < 0.001) (Figure 7C). The post hoc tests for multiple comparisons showed a significant increase in the mRNA levels of *Ppara* in the non-stress alcohol subgroup compared with the non-stress saline subgroup (*** *q* < 0.001), and a significant decrease in the stress alcohol subgroup compared with the non-stress saline (*** *q* < 0.001), non-stress alcohol (^###^ *q* < 0.001) and stress saline (^$$$^ *q* < 0.001) subgroups. In addition, the expression of *Ppara* was significantly increased in the stress saline subgroup compared with the non-stress saline subgroup (* *q* < 0.05).

#### 3.6.2. Enzymes of Synthesis

- As shown in Figure 7D, the statistical analysis revealed one main effect of repeated restraint stress on the mRNA levels of *Napepld* (f1: *F*_1,33_ = 41.06; *p* < 0.001) and a significant interaction between both factors (f1 × f2: *F*_1,33_ = 39.01; *p* < 0.001). The post hoc tests for multiple comparisons showed a significant increase in the expression of *Napepld* in the non-stress alcohol subgroup compared with the non-stress saline subgroup (*** *q* < 0.001), and a significant decrease in the stress alcohol subgroup compared with the non-stress saline (*** *q* < 0.001), non-stress alcohol (^###^ *q* < 0.001) and stress saline (^$$$^ *q* < 0.001) subgroups.
- Regarding the expression of *Dagla*, there was a significant main effect of repeated restraint stress (f1: *F*_1,33_ = 4.45; *p* = 0.043) and a significant interaction between both factors (f1 × f2: *F*_1,33_ = 10.13; *p* = 0.003) (Figure 7E). The multiple comparisons showed a significant increase in the expression of *Dagla* in the non-stress alcohol subgroup compared with the non-stress saline subgroup (* *q* < 0.05). In contrast, there was a significant decrease of *Dagla* in the stress alcohol subgroup compared with the non-stress alcohol subgroup (^##^ *q* < 0.01).
- The statistical analysis of *Daglb* revealed significant main effects of repeated restraint stress (f1: *F*_1,33_ = 57.34; *p* < 0.001) and adolescent alcohol exposure (f2: *F*_1,33_ = 44.84; *p* < 0.001), but also a significant interaction (f1 × f2: *F*_1,33_ = 109.2; *p* < 0.001) (Figure 7F). The post hoc tests showed a significant increase in the expression of *Daglb* in the non-stress alcohol subgroup compared with the non-stress saline subgroup (*** *q* < 0.001), and a significant decrease in the stress alcohol subgroup compared with the non-stress alcohol (^###^ *q* < 0.001) and stress saline (^$^ *q* < 0.05) subgroups.

#### 3.6.3. Enzymes of Degradation

- A two-way ANOVA revealed significant main effects of repeated restraint stress (f1: *F*_1,32_ = 11.19; *p* = 0.002) and adolescent alcohol exposure (f2: *F*_1,32_ = 59.38; *p* < 0.001) on the mRNA levels of *Faah*, but no significant interaction between factors (Figure 7G). Rats exposed to restraint stress had significantly lower levels of *Faah* than non-stressed rats, and rats exposed to intermittent alcohol had significantly lower levels of *Faah* than rats with saline.
- The statistical analysis of the gene expression of MAGL revealed one significant main effect of repeated restraint stress (f1: *F*_1,33_ = 134.0; *p* < 0.001) and a significant interaction between both factors (f1 × f2: *F*_1,33_ = 267.4; *p* < 0.001) on *Mgll* (Figure 7H). The post hoc tests showed a significant increase in the mRNA levels of *Mgll* in the non-stress alcohol (*** *q* < 0.001) and stress saline (** *q* < 0.01) subgroups compared with the non-stress saline subgroup, and a significant decrease in the stress alcohol subgroup compared with the non-stress saline (*** *q* < 0.001), non-stress alcohol (^###^ *q* < 0.001) and stress saline (^$$$^ *q* < 0.001) subgroups.

### 3.7. Effects of Restraint Stress and Intermittent Alcohol on the mRNA Expression of Glutamatergic-Signaling Genes in the mPFC

Finally, we evaluated the effects of repeated restraint stress and intermittent alcohol exposure during adolescence on the mRNA expression of some receptors involved in the glutamatergic signaling in the mPFC.

#### 3.7.1. Metabotropic Receptor

**Figure 8:**
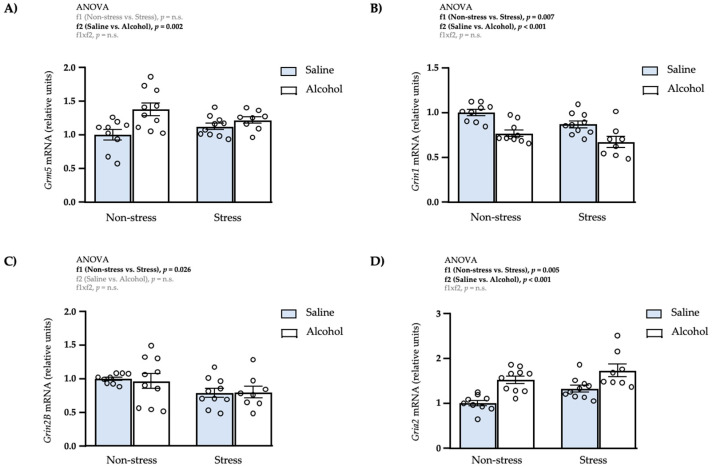
Relative mRNA expression levels of glutamatergic receptor subunits in the mPFC of adult male rats after repeated restraint stress and/or intermittent alcohol exposure during adolescence. Relative mRNA expression of *Grm5* (**A**); *Grin1* (**B**); *Grin2B* (**C**); and *Gria2* (**D**) in male adult rats after repeated restraint stress (f1) and/or intermittent alcohol exposure (f2) during adolescence. Bars represent the mean ± SEM (8–10 rats/subgroup). Data were analyzed by two-way ANOVA. *p*-values in bold denote significant main effects of factors (f1 and f2).

As shown in Figure 8A, a two-way ANOVA revealed one significant main effect of adolescent alcohol exposure (f2: *F*_1,33_ = 11.21; *p* = 0.002) on the mRNA levels of *Grm5*. Rats exposed to intermittent alcohol had significantly higher levels of *Grm5* than rats with saline.

#### 3.7.2. NMDA Receptors

- The statistical analysis revealed significant main effects of repeated restraint stress (f1: *F*_1,33_ = 8.15; *p* = 0.007) and adolescent alcohol exposure (f2: *F*_1,33_ = 28.47; *p* < 0.001) on the mRNA expression of *Grin1* (Figure 8B). Thus, rats exposed to restraint stress had significantly lower levels of *Grin1* than non-stressed rats, and rats exposed to intermittent alcohol had also significantly lower levels of *Grin1* than rats with saline.
- Regarding the mRNA expression of *Grin2B*, there was a significant main effect of repeated restraint stress (f1: *F*_1,32_ = 5.46; *p* = 0.026) and rats exposed to stress had significantly lower levels of *Grin2B* than non-stressed rats (Figure 8C).

#### 3.7.3. AMPA Receptor

A two-way ANOVA revealed significant main effects of repeated restraint stress (f1: *F*_1,33_ = 9.30; *p* = 0.005) and adolescent alcohol exposure (f2: *F*_1,33_ = 26.98; *p* < 0.001) on the mRNA levels of *Gria2*, but no significant interaction (Figure 8D). Rats exposed to restraint stress had significantly higher levels of *Gria2* than non-stressed rats, and rats exposed to intermittent alcohol had also significantly higher levels of *Gria2* than rats with saline.

## 4. Discussion

We have recently reported that both an acute restraint stress episode and excessive alcohol consumption during adolescence result in an adult phenotype characterized by anxiety-like behaviors and high plasma levels of corticosterone, but this common anxious phenotype is associated with different disturbances in the amygdala circuits [31].

Interestingly, after the repeated restraint stress, the rats exposed to restraint stress displayed lower body weight gain than the non-stressed rats. In agreement with that, previous studies have reported stress-induced reductions in body weight gain in rodents [35,36,37,38]. Changes in body weight gain may be considered as an index of the physiological effects of the stressor; in fact, there was a recovery after finishing the restraint period, and differences in body weight gain were not observed during alcohol exposure between the stressed and non-stressed rats in the following weeks. In the present study, we have investigated the long-term effects of repeated restraint stress and intermittent alcohol exposure during adolescence on anxiety-like behaviors and the mRNA expression of the main components of the relevant signaling systems involved in the modulation of reward and anxiety/stress responses in the amygdala and the mPFC of male Wistar rats.

In adulthood (PND76), all groups of rats underwent behavioral testing on the EPM. We found that chronic restraint stress or intermittent alcohol exposure resulted in increased anxiety-like behaviors compared with control rats, but no differences in spontaneous motor activity. These results support the idea that adolescent stress as well as excessive alcohol consumption can have enduring effects and can shape future behaviors, resulting in a high-anxiety phenotype in adulthood. Our results are consistent with previous reports of increased anxiety-like behaviors in rats exposed to chronic restraint stress [10,39,40] or excessive alcohol consumption [31,33,41], and the combination of stress and alcohol did not induce higher anxiogenesis than those produced by chronic stress or alcohol individually, which suggests a ceiling effect [31]. While anxiety-like behaviors were associated with higher plasma corticosterone levels in rats exposed to alcohol, we did not observe alterations in this stress hormone in rats exposed to chronic restraint stress. By contrast, we have previously described that acute restraint stress induces a long-term increase in plasma corticosterone levels [31]. The present results may suggest a habituation of the neuroendocrine response after chronic restraint stress. In this regard, previous studies have reported that the corticosterone response is blunted in rodents after repeated exposure to the same stressor [42,43], and the exposure to chronic homotypic stressors given at predictable intervals may produce habituation [8]. Moreover, adolescent rodents exposed to a chronic stress display a higher corticosterone level immediately after cessation of the stressor, but a rapid recovery to baseline levels [44]. 

During adolescence, many brain areas are known to undergo functional and structural reorganization. Among the primary structures involved in stress, emotional regulation, motivation and reward, are the mPFC and the amygdala, which are vulnerable to the effects of chronic stress and alcohol exposure [45]. The ECS is widely expressed in both brain regions [46], and this system is also undergoing dynamic changes during adolescence [47,48]. Thus, some alterations of these processes of maturation during this crucial period may result in enduring consequences, including alterations in emotional behaviors and stress sensitivity.

### 4.1. Amygdala

Previous studies from our group have described a general deficit in the gene expression of the ECS in the amygdala of rats exposed to alcohol during adolescence [33,41]. In accordance with these previous studies, the present results showed similar neuroadaptations of the ECS in response to an intermittent alcohol exposure during adolescence. However, we observed an upregulation of the mRNA expression of *Cnr2* that may be associated with alcohol-induced neuroinflammation [49]. In fact, CB2R receptors are found in microglia and astrocytes in the CNS [50], and these receptors are upregulated under neuroinflammatory conditions [51]. Rats exposed to early repeated restraint stress also displayed a general deficit in the gene expression of the amygdalar ECS, but an increase in the mRNA expression of *Cnr2*. Numerous studies have reported the effects of the stress on the ECS [for review see [20]], but these effects depend on many factors, including the brain region, the type of stressor or the chronicity of the stress. Finally, the combination of repeated restraint stress and alcohol consumption did not produce a synergism effect on the mRNA expression of the main components of the ECS in the amygdala, which suggests an overall ceiling effect. The present results suggest that adolescent alcohol exposure and an early repeated stress produce long-term changes in the gene expression of the main components of the ECS. These changes could produce neuroadaptive modifications in amygdalar endocannabinoid signaling that may be associated with the development of negative responses, such as anxiety-like behaviors. However, a main limitation of this study is that we have not yet examined the protein expression of the different components of the ECS, and it is difficult to ascertain whether the alterations in the mRNA expression are directly related to changes in protein levels or enzymatic activity, given that there are numerous posttranscriptional and translational mechanisms that would need to be considered. Moreover, because endocannabinoids act as retrograde messengers, the mRNA transcripts and the proteins are not localized in the same brain nuclei in projection neurons. Therefore, the association between changes in the gene expression and the protein expression, and activity of these enzymes should be addressed in future research.

For a better understanding of the mechanisms underlying the long-term effects of repeated restraint stress and/or adolescent alcohol exposure, we also evaluated the alterations of the gene expression of some glutamate receptor subunits in the amygdala of these animals. Overall, we found that both stress and alcohol produced changes in the mRNA expression of glutamate receptor subunits. In agreement with our previous report [31], the present results provide new evidence of the hyperactivity of glutamatergic signaling in the amygdala that may be associated with an inefficient inhibitory function of the ECS and may contribute to the anxious phenotype exhibited by these animals. Interestingly, there were significant interaction effects between repeated restraint stress and alcohol exposure on the gene expression of the subunits of NMDA receptors, with decreased mRNA expression of *Grin1* and increased mRNA expression of *Grin2B*. The NMDA receptors are involved in numerous physiological and pathophysiological processes, and variations in the subunit composition may affect their function [52]. Therefore, our results suggest that the combination of an early repeated restraint stress and alcohol exposure during adolescence might modify the glutamate transmission through alterations in the composition of NMDA receptors.

Because both CRH and NPY signaling systems are involved in the regulation of the stress responses, we also analyzed their mRNA expression in the amygdala of these rats. We found that alcohol exposure induced a decrease in the mRNA expression of *Crh*, but an increase in the mRNA expression of its receptor. As mentioned previously, these alterations in the gene expression may not be related to similar changes in protein levels. However, these results suggest an increase in the signaling mediated by CRH that may be associated to anxiogenic-like behaviors. In agreement with that, we have previously described an increase in the protein expression of CRHR1 in the amygdala of rats exposed to alcohol during adolescence [31]. Regarding the NPY system, we found a decrease in the mRNA levels of *Npy* and its receptor in alcohol-exposed rats. This decrease may also be associated to the anxiety state exhibited by these rats [33]. Unlike alcohol exposure, repeated restraint induced a slight decrease in the mRNA expression of *Crh*, *Crhr1* and *Npy*, but no changes on the mRNA levels of *Npy1r*. Moreover, the combination of stress and alcohol did not produce different alterations to those produced by stress or alcohol alone. These results provide new evidence of the existence of different mechanistic pathways involved in the anxious phenotype associated with stress and/or alcohol exposure.

### 4.2. Medial Prefrontal Cortex

We have previously reported a general potentiation of endocannabinoid signaling in rats exposed to alcohol during adolescence in the mPFC, [33,41]. This is supported by the present results because the mRNA expression of many components of the ECS was increased in non-stressed rats exposed to alcohol in comparison with control rats two weeks after the last alcohol exposure. These results suggest a long-term potentiation of endocannabinoid signaling in the mPFC by increasing the production of endocannabinoids, which might be associated with the homeostatic role of this system in response to the withdrawal-induced negative-affective states [20,53,54]. In addition, the increase in the mRNA expression of *Ppara* and *Napepld* also suggests an increase in the signaling mediated by other non-cannabinoid N-acylethanolamines (e.g., oleoylethanolamide and palmitoylethanolamide) that are involved in neuroprotection [55,56]. Contrarily to the effects observed in rats exposed to alcohol without restraint stress, we did not find many significant alterations on the mRNA expression of the ECS in the mPFC of young adult rats exposed to early repeated restraint stress. Interestingly, we observed that the combination of stress and alcohol produced different effects to those produced by stress or alcohol separately. In the present study, rats exposed to stress and alcohol displayed a decrease in the mRNA expression of the main components of the ECS in the mPFC. These long-term alterations in the endocannabinoid signaling might be relevant in the modulation of reward and behavioral processes. For example, the ECS plays a protective role against the development of anhedonia after chronic stress [20], therefore a deficiency in endocannabinoid signaling after a combination of stress and alcohol may result in an impairment to reward sensitivity or anhedonia.

Finally, we also found that both stress and alcohol produced changes in the mRNA expression of glutamate receptor subunits in this brain region. However, we observed that the combination of repeated restraint stress and alcohol consumption did not produce different effects on the glutamatergic system than those produced by chronic stress or alcohol individually.

## 5. Conclusions

In summary, the present data demonstrated that both repeated restraint stress and alcohol exposure during adolescence produced long-term anxiety-like behaviors. However, while rats exposed to intermittent alcohol displayed high plasma corticosterone levels, we did not observe alterations of this hormone in the stressed animals, which suggests habituation after repeated restraint stress. Moreover, we have also demonstrated that these long-term effects on emotional behaviors were associated with different alterations in the mRNA expression of proteins related to the ECS and other signaling systems, such as the glutamatergic, CRH and NPY systems, in the amygdala and the mPFC (an overall summary is shown in Table S2). The present study provides new evidence of the existence of partially different mechanisms by which an early repeated stress, adolescent alcohol exposure or the combination of both lead to maladaptive changes in emotional behaviors. However, we are aware that this study has an important limitation because female rats were not included. Therefore, further investigation is necessary to elucidate sex differences in the mechanisms that regulate stress responses and anxiety-like behaviors after early exposures to stressful events.

## Data Availability

Not applicable.

## Supplementary Materials

The following supporting information can be downloaded at: https://www.mdpi.com/article/10.3390/biomedicines10030593/s1, Table S1: Primer references for TaqMan Gene Expression Assays (Applied Biosystems). Table S2: Summary of changes in the gene expression of components of the endocannabinoid, glutamatergic, CRH and NPY systems in the amygdala and the mPFC of male rats exposed to restraint stress and/or intermittent alcohol during adolescence.
