# Supplementary material for: Repeated Restraint Stress and Binge Alcohol during Adolescence Induce Long-Term Effects on Anxiety-like Behavior and the Expression of the Endocannabinoid System in Male Rats
Source: Biomedicines. 2022 Mar 3;10(3):593. doi: 10.3390/biomedicines10030593 (PMC8945821; doi:10.3390/biomedicines10030593)
Supplement: Supplementary file 1 [file biomedicines-10-00593-s001.zip › biomedicines-1602051-supplementary.pdf]

**Table S1.** Primer references for TaqMan® Gene Expression Assays (Applied Biosystems).

| **Gene description** | **Assay ID** | **No accession GenBank** | **Amplicon Length** |
|---|---|---|---|
| ***Actb*** | Rn00667869_m1 | NM_031144.3 | 91 |
| ***Cnr1*** | Rn02758689_s1 | NM_012784.4 | 92 |
| ***Cnr2*** | Rn01637601_m1 | NM_020543.4 | 68 |
| ***Dagla*** | Rn01454304_m1 | NM_001005886.1 | 67 |
| ***Daglb*** | Rn01453771_m1 | NM_001107120.1 | 98 |
| ***Mgll*** | Rn00593297_m1 | NM_138502.2 | 78 |
| ***Napepld*** | Rn01786262_m1 | NM_199381.1 | 71 |
| ***Faah*** | Rn00577086_m1 | NM_024132.3 | 63 |
| ***Ppara*** | Rn00566193_m1 | NM_013196.1 | 98 |
| ***Crh*** | Rn01462137_m1 | NM_031019.1 | 112 |
| ***Crhr1*** | Rn00578611_m1 | XM_006247542.2 | 58 |
| ***Npy*** | Rn00561681_m1 | NM_012614.2 | 63 |
| ***Npy1r*** | Rn02769337_s1 | NM_001113357.1 | 98 |
| ***Grm5*** | Rn00566628_m1 | NM_017012.1 | 112 |
| ***Grin1*** | Rn01436034_m1 | NM_001270602.1 | 73 |
| ***Grin2b*** | Rn00680474_m1 | NM_012574.1 | 79 |
| ***Gria2*** | Rn00568514_m1 | NM_001083811.1 | 122 |

**Table S2.** Summary of changes in the gene expression of components of the endocannabinoid, glutamatergic, CRH and NPY systems in the amygdala and the mPFC of male rats exposed to restraint stress and/or intermittent alcohol during adolescence.

| **Signaling systems (genes)** | **AMYGDALA*** | | | **mPFC*** | | |
|---|---|---|---|---|---|---|
| | **Stress** | **Alcohol** | **Stress+Alcohol** | **Stress** | **Alcohol** | **Stress+Alcohol** |
| **ECS** | | | | | | |
| ***Cnr1*** | ▼ | ▼ | ▼ | ⟝ | ▲ | ▼ |
| ***Cnr2*** | ▲ | ▲ | ▲ | ⟝ | ↓ | ▼ |
| ***Ppara*** | ⟝ | ▼ | ▼ | ▲ | ▲ | ▼ |
| ***Napepld*** | ↓ | ▼ | ▼ | ⟝ | ▲ | ▼ |
| ***Dagla*** | ⟝ | ⟝ | ↓ | ⟝ | ▲ | ⟝ |
| ***Daglb*** | ▼ | ▼ | ▼ | ⟝ | ▲ | ⟝ |
| ***Faah*** | ▼ | ▼ | ▼ | ⟝ | ▼ | ▼ |
| ***Mgll*** | ▼ | ▼ | ▼ | ▲ | ▲ | ▼ |
| **Glutamate** | | | | | | |
| ***Grm5*** | ⟝ | ▲ | ⟝ | ⟝ | ▲ | ↑ |
| ***Grin1*** | ⟝ | ⟝ | ▼ | ↓ | ▼ | ▼ |
| ***Grin2B*** | ⟝ | ⟝ | ▲ | ↓ | ⟝ | ↓ |
| ***Gria2*** | ↑ | ↑ | ↑ | ↑ | ▲ | ▲ |
| **CRH** | | | | | | |
| ***Crh*** | ↓ | ▼ | ▼ | X | X | X |
| ***Crhr1*** | ↓ | ▲ | ▲ | | | |
| **NPY** | | | | | | |
| ***Npy*** | ↓ | ▼ | ▼ | X | X | X |
| ***Npyr1*** | ⟝ | ▼ | ⟝ | | | |

(*) The direction of the changes was determined in relation to the non-stress saline subgroup in the amygdala and the mPFC. Symbols: (▼▲) = significant difference, (↓↑) = tendency, (⟝) = no change, (X) = not determined.
